# Preparation and Evaluation of Antimicrobial Property and Anti-inflammatory Activity of Fenugreek Gel Against Oral Microbes: An Invitro Study

**DOI:** 10.7759/cureus.47659

**Published:** 2023-10-25

**Authors:** Vyshnavi B Sindhusha, Arvina Rajasekar

**Affiliations:** 1 Periodontics, Saveetha Dental College and Hospitals, Saveetha Institute of Medical and Technical Sciences, Saveetha University, Chennai, IND

**Keywords:** periodontitis, anti-inflammatory activity, antimicrobial activity, local drug delivery, fenugreek seeds

## Abstract

Aim

Periodontal dressings play a crucial role in periodontal surgery and dental procedures. These dressings have several functions and benefits, similar to surgical wound dressings used in other surgical contexts. Periodontal dressings protect the surgical site and control bleeding in the oral cavity as they exert pressure on tissue and blood vessels. By protecting the wound and stabilizing the tissues, periodontal dressings create an environment that encourages proper and faster healing. Recently, the use of periodontal pack has reported various postoperative discomfort to the patients. This led to the development of an interest in considering fenugreek as an alternative to periodontal dressing as it possesses various antidiabetic and hypolipidemic effects. Thus, fenugreek can be used as an alternative to periodontal dressing. The study aimed to prepare and evaluate the antimicrobial nature of fenugreek gel in oral microbes and the anti-inflammatory properties of gel with protein coagulation in egg albumin.

Materials and methods

The fenugreek gel preparation was done by grinding 100 g of fenugreek seeds into a powder and adding 100 ml of distilled water to the powder and then heating the mixture at 70°C for 30 minutes. Five milliliters of the fenugreek concentrate were added to an equal mixture of carboxymethyl cellulose and Carbopol which was mixed thoroughly to form a gel. The antimicrobial nature of fenugreek has been evaluated in various organisms such as *Streptococcus mutans, Lactobacillus, Enterococcus faecalis, and Candida albicans* whereas the anti-inflammatory property was evaluated by protein coagulation method in egg albumin.

Results

The results stated that the fenugreek gel at a concentration of 100 µg/ml showed a greater zone of inhibition (5.39 ± 0.05) compared with doxycycline (1.1 ± 0.08) for a high antimicrobial potential against all oral microbes. The anti-inflammatory activity of the gel by protein coagulation method in egg albumin showed greater inhibition (67.15±1.36) at 100 µg/ml of fenugreek extract when compared with aspirin (64.43±2.93). Paired t-test was done for both the properties and the p-value was less than 0.5 stating that the difference between the groups was statistically significant.

Conclusion

The present study showed that the fenugreek gel possesses higher antimicrobial and anti-inflammatory properties when compared with doxycycline and aspirin, respectively. Hence, fenugreek gel can be used as an alternative periodontal dressing to reduce postoperative inflammation.

## Introduction

Periodontium is a critical component of the oral cavity, playing a vital role in maintaining the teeth’s health and stability. The periodontal ligament (PDL) is a specialized connective tissue that consists of collagen fibers and other components. The PDL’s primary functions include the barrier for the gingival tissues, the junctional epithelium, and the connective tissue attachment, collectively helping to prevent infections and maintain oral health [1].

Periodontitis is an inflammatory disease that occurs in response to plaque accumulation on the tooth surface. These biofilms contain harmful bacteria that trigger an immune response, leading to inflammation in the surrounding tissues [2]. The initial step for periodontal treatment includes the removal of bacterial biofilms through non-surgical procedures. Resective therapies involve surgical procedures that remove damaged tissue and reshape the bone, improving the functional efficiency of periodontium [3]. Reconstructive therapies are designed to resolve inflammation and restore the form and function of the periodontium, promoting long-term periodontal health [4]. Periodontal wound healing includes key phases of wound healing such as hemostasis and coagulation, the initial phase of wound healing; inflammation; cell proliferation and wound remodeling; and maturation, leading to remodeling of the newly formed tissue. Collagen fibers are reorganized and strengthened, and excess collagen is broken down. This phase aims to restore tissue strength and function [5]. Activation of inflammatory cells such as polymorphonuclear neutrophils and monocytes plays a crucial role in the wound healing process [6]. Surgical dressing helps prevent wound contamination and aids post-surgical healing. The primary goals of dressings are to protect the surgical site, stabilize soft tissues, immobilize mobile teeth, reduce tooth hypersensitivity, and enhance patient comfort. The periodontal dressings have evolved in response to research findings and changes in dental materials [7]. New dressing formulations aim to provide the desired protective and stabilizing benefits without causing allergic reactions or discomfort. The choice of surgical dressing can significantly affect patient comfort and the overall success of periodontal surgery [8].

Periodontal packs can serve as a delivery system for therapeutic agents directly onto the affected periodontal tissues; they also have antimicrobial agents to target oral microbes in the surgical site [9]. The fenugreek (*Trigonella foenum-graecum*) plant is known for its various properties and has a history of traditional use in Asia and Mediterranean countries [10]. Fenugreek contains numerous bioactive compounds, including alkaloids, tannins, flavonoids, glycosides, and terpenoids. These compounds may explain its potential antimicrobial activity [11]. The antimicrobial properties of fenugreek can contribute to phytomedicine, where natural remedies and plant-derived compounds are used in the treatment of various health ailments such as hyperglycemia, hyperlipidemia, and gastrointestinal upset [12]. Hence, fenugreek could be used as an alternative periodontal dressing to reduce postoperative inflammation.

This study represents the preparation and evaluation of the antimicrobial and anti-inflammatory properties of fenugreek gel. The antimicrobial activity was tested against selected microorganisms such as *Streptococcus mutans*, *Lactobacillus*, *Enterococcus faecalis*, and *Candia albicans*. Anti-inflammatory activity was tested with the protein coagulation method in egg albumin.

## Materials and methods

**Figure 1:**
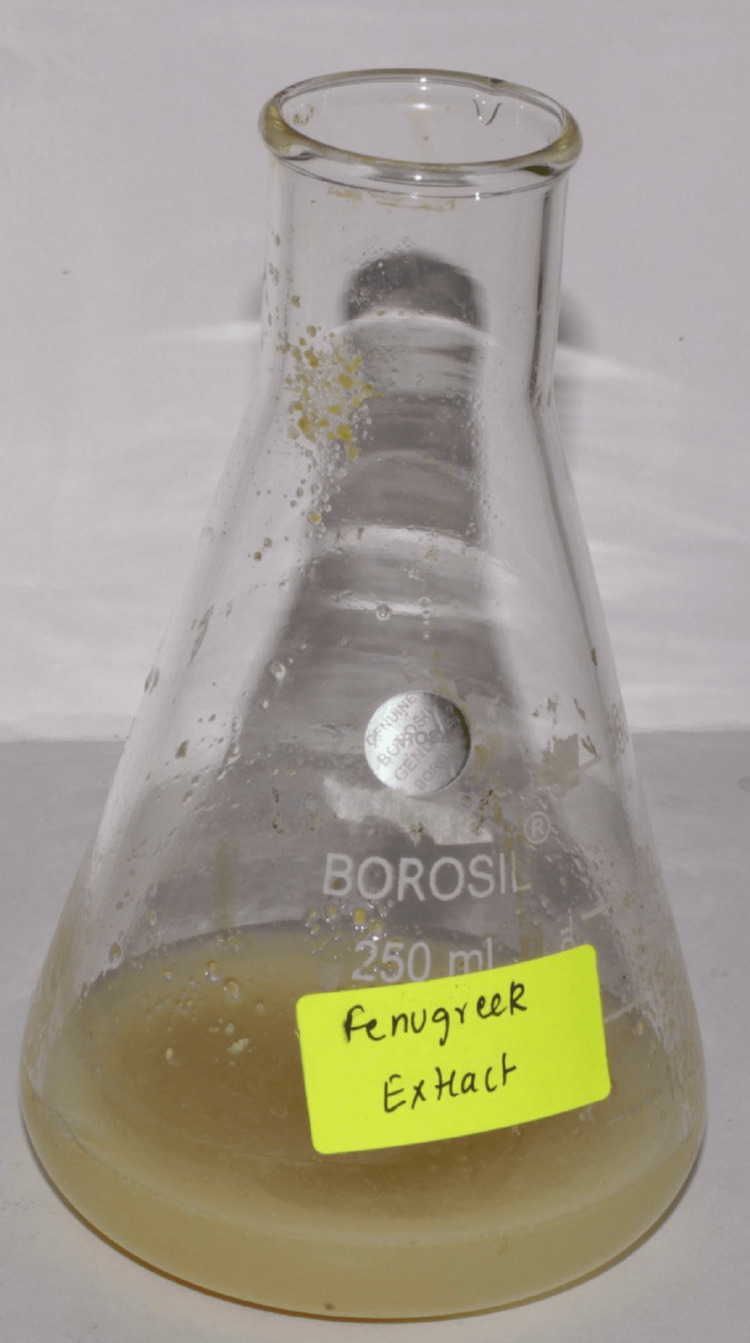
Fenugreek extract The fenugreek extract was been prepared by grinding 5 g of commercially available fenugreek seeds and adding 100 ml distilled water, then heating the solution at 70°C for 30 minutes.

After obtaining approval from the scientific board of Saveetha Dental College (SRB/SDC/PERIO-2104/23/073). A hundred micrograms of commercially available fenugreek seeds were ground into a powder. Five grams of fenugreek powder was added to 100 ml of distilled water and boiled at a temperature of 70°C (158°F) for 30 minutes. This helps in extracting active compounds from the fenugreek seeds and a thick extract formation (Figure 1).

**Figure 2:**
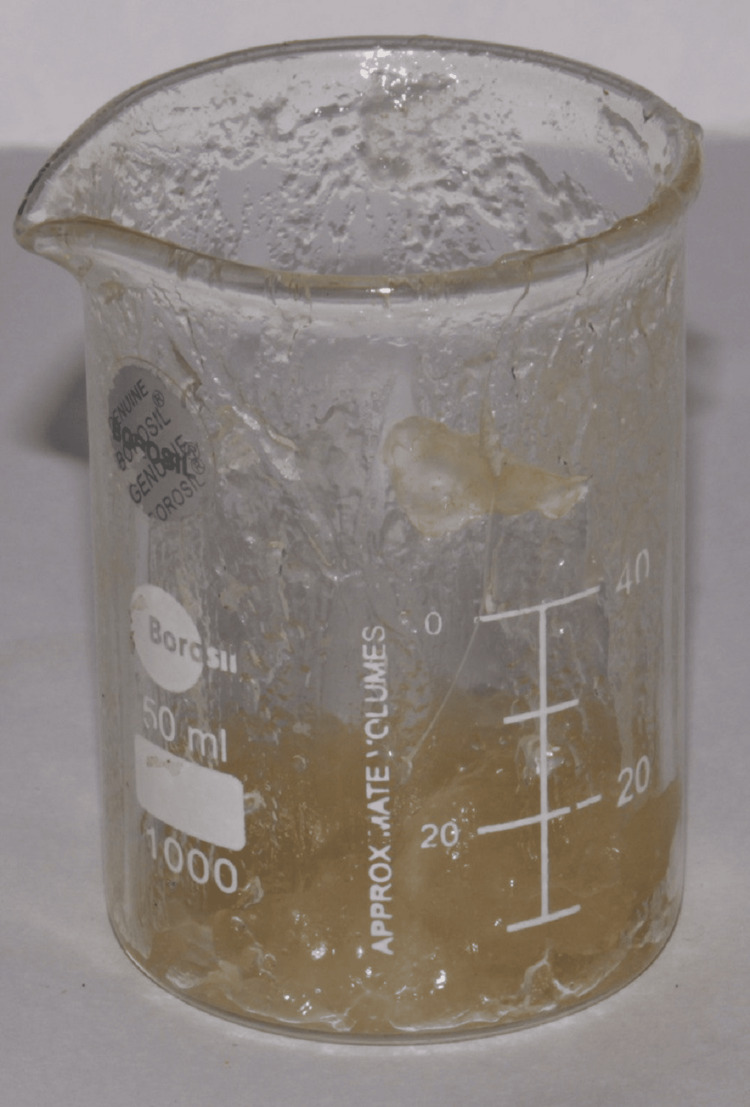
Fenugreek gel The gel preparation was done by adding 5 ml of concentrated fenugreek extract to the Carbopol and carboxymethyl cellulose mixture.

Simultaneously, 3 g of an equal mixture was created from Carbopol (1.5 g) and carboxymethylcellulose (1.5 g) to which 20 ml of distilled water was added to make a thick mixture that provides the desired gel-like consistency. Five milliliters of the concentrated fenugreek extract was added to this thick mixture which led to the formation of fenugreek gel (Figure 2).

Antimicrobial activity

The antimicrobial activity of fenugreek extract was tested using the agar well diffusion method [13]. One gram of fenugreek gel was diluted into different concentrations: 25 µg; 50 µg; and 100 µg. Doxycycline gel was used as a control (as it inhibits bacterial protein synthesis by allosterically binding to the 30S prokaryotic ribosomal subunit). These gel dilutions were then inoculated onto agar plates, which are used for microbial assays. Each prepared microorganism, that is, *S. mutans*, *Lactobacillus*, *E. faecalis*, and *C. albicans* were inoculated onto separate agar plates. The inoculated agar plates were incubated in a controlled environment and examined for the presence or absence of microbial growth. The maximum antimicrobial growth of fenugreek gel was observed against *C. albicans* followed by *S. mutans* with a minimum inhibitory concentration value of 100 mg/ml [14]. The maximum antimicrobial activity of doxycycline is observed in *E. faecalis* followed by *C. albicans* [15].

Anti-inflammatory activity

The anti-inflammatory activity was assessed by the protein-denaturation method, using a reaction mixture (5 ml) consisting of 0.2 ml of egg albumin (from fresh hen’s egg), 2.8 ml of phosphate-buffered saline (PBS, pH 6.4), and 2 ml of varying concentrations of fenugreek extract (25, 50, and 100 μg/ml). An equal volume of aspirin (2.5 ml) and distilled water (2.5 ml) was used as control. The mixtures were incubated at 37°C in a biological oxygen demand incubator (Lab-line Technologies) for 15 min and then heated at 70°C for 5 min. After cooling, their absorbance was measured.

Statistical analysis

Paired t-test was done in both antimicrobial and anti-inflammatory tests. The p-value was less than 0.5 stating that the difference between the groups was statistically significant.

## Results

Antimicrobial activity

**Figure 3:**
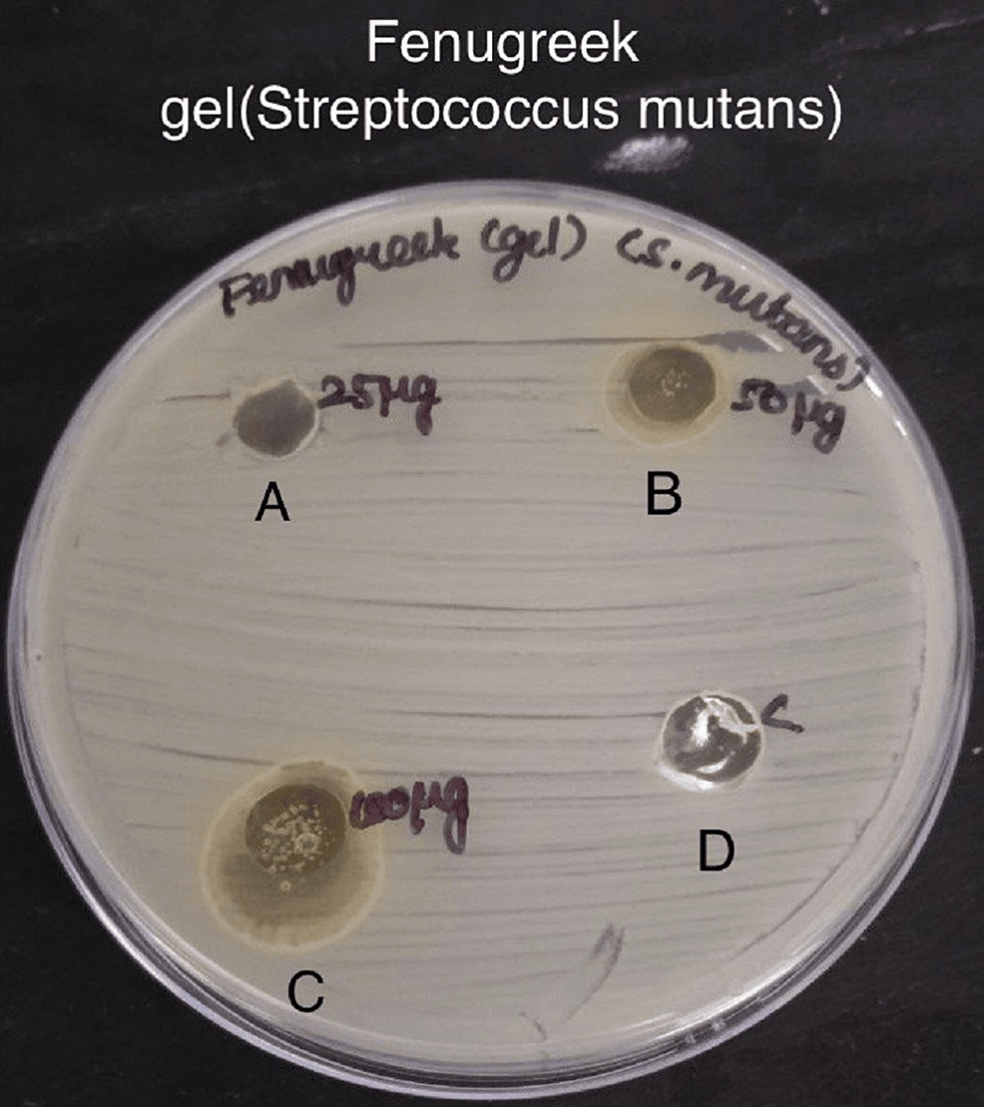
Antimicrobial activity of fenugreek gel against Streptococcus mutans The antimicrobial activity is seen against *S. mutans* in agar medium with A having 25 µg concentration of fenugreek gel, B having 50 µg, C having 100 µg concentration, and D is the control which is doxycycline.

**Figure 4:**
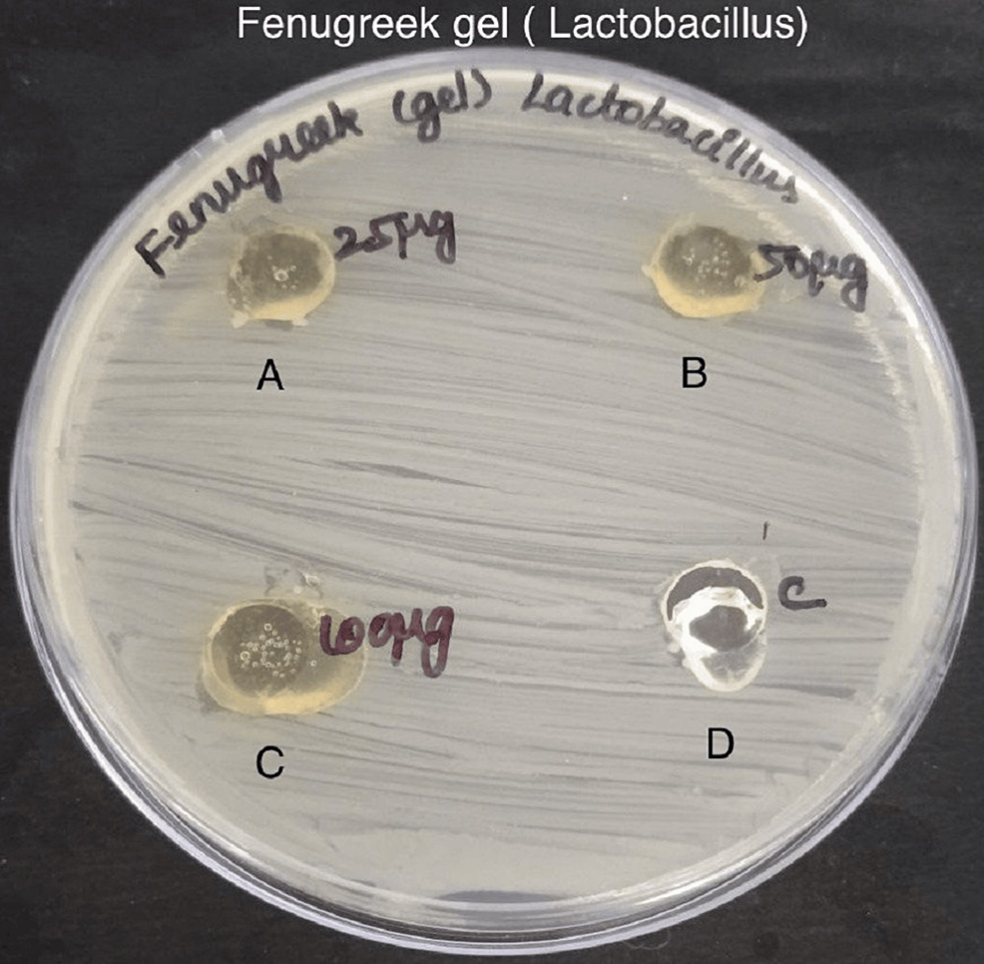
Antimicrobial activity of fenugreek gel against Lactobacillus The antimicrobial activity is seen against *Lactobacillus *in agar medium with A having 25 µg concentration of fenugreek gel, B having 50 µg concentration, C having 100 µg concentration, and D is the control which is doxycycline.

**Figure 5:**
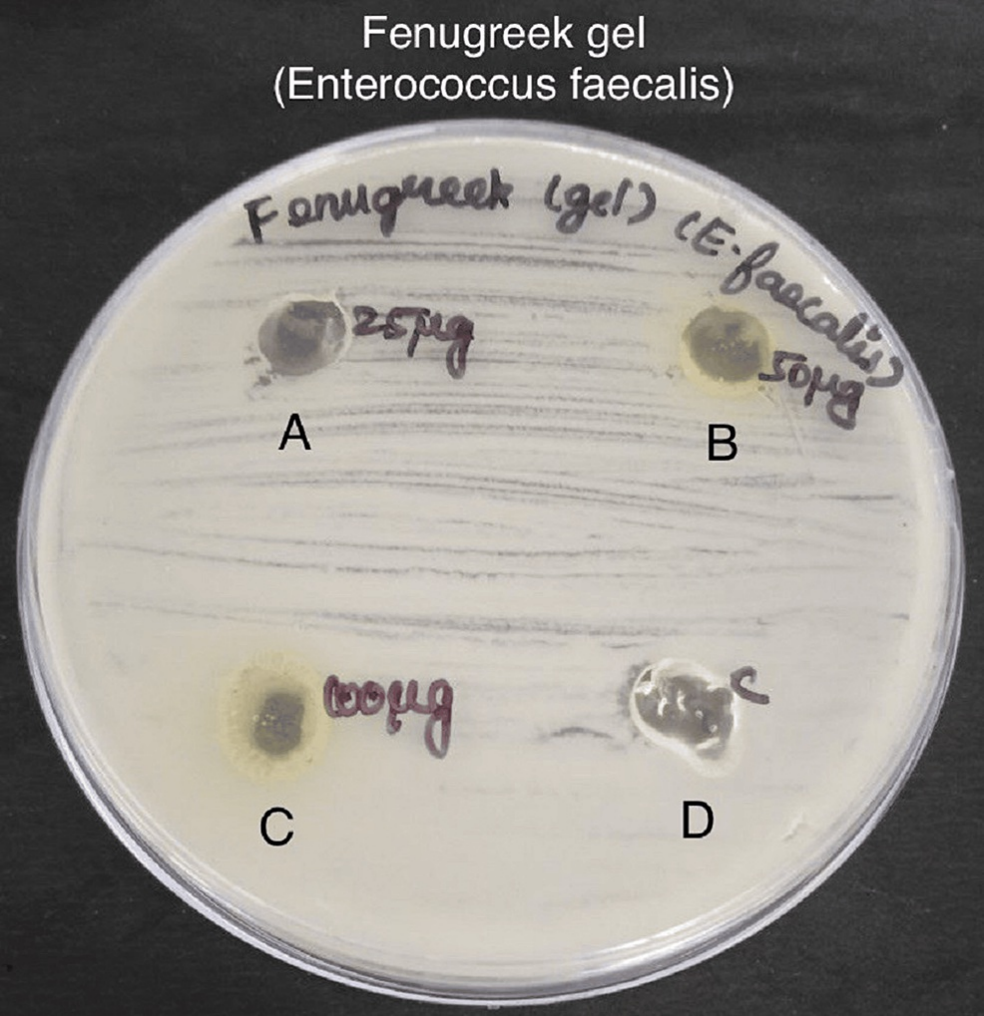
Antimicrobial activity of fenugreek gel against Enterococcus faecalis The antimicrobial activity is seen against *E. faecalis* in agar medium with A having 25 µg concentration of fenugreek gel, B having 50 µg concentration, C having 100 µg concentration, and D is the control which is doxycycline.

**Figure 6:**
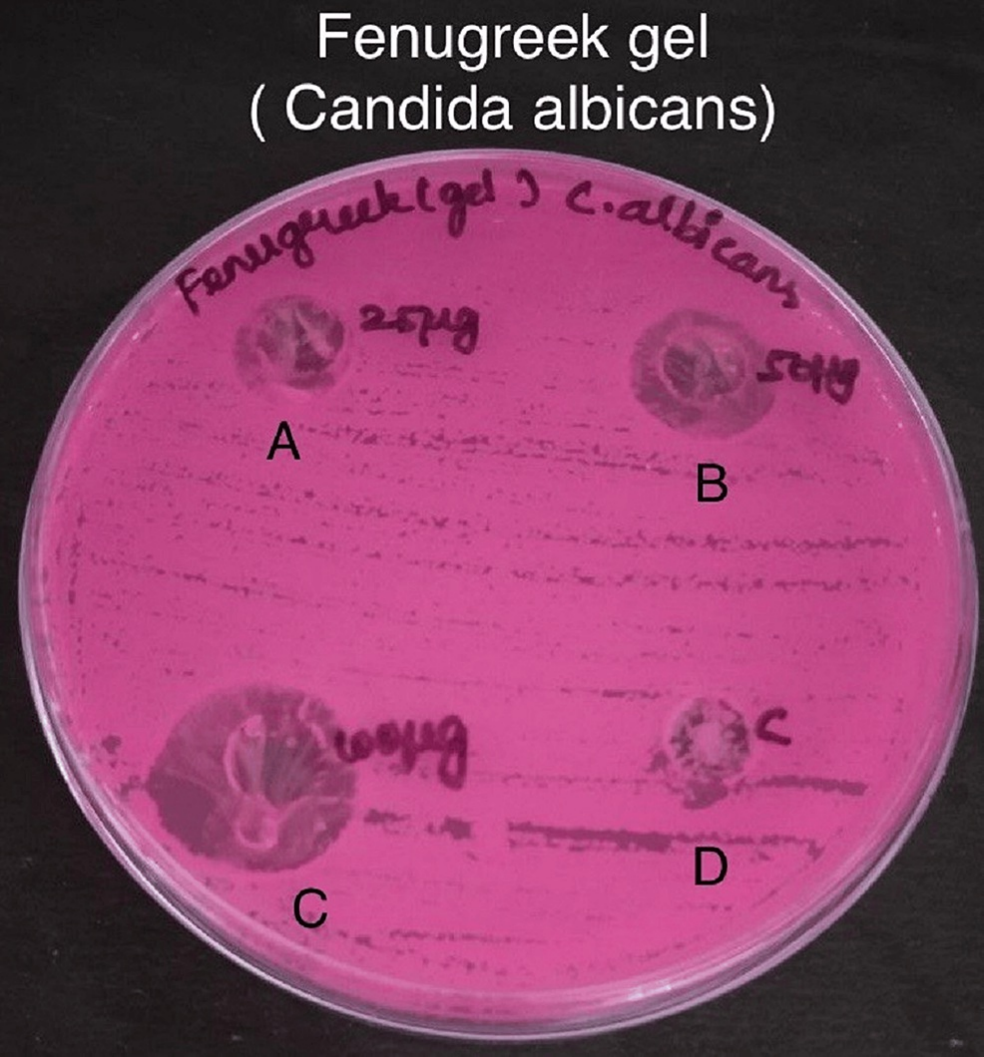
Antimicrobial activity of fenugreek gel against Candida albicans The antimicrobial activity is seen against *C. albicans* in agar medium with A having 25 µg concentration of fenugreek gel, B having 50 µg concentration, C having 100 µg concentration, and D is the control which is doxycycline.

Antimicrobial activity was assessed by measuring the zone of inhibition in the experimental and control groups against specific bacteria - *S. mutans*, *Lactobacillus*, *E. faecalis*, and *C. albicans* in Figures 3-6.

The mean in the widths of the zones of inhibition for different concentrations of fenugreek gel and doxycycline in agar plates inoculated with *S. mutans*, *Lactobacillus*, *E. faecalis*, and *C. albicans* are listed in Table 1.

**Table 1:** The mean and standard deviation in the widths of the zones of inhibition among different microorganisms. The widths of the zones of inhibition for different concentrations of fenugreek gel and doxycycline in various microorganisms such as *S. mutans*, *Lactobacillus*, *E. faecalis*, and *C. albicans*.

Different concentrations of fenugreek gel	Streptococcus mutans	Lactobacillus	Enterococcus faecalis	Candida albicans
	[Zone of Inhibition(mm) ± Standard Deviation(mm)]	[Zone of Inhibition(mm) ± Standard Deviation(mm)]	[Zone of Inhibition(mm) ± Standard Deviation(mm)]	[Zone of Inhibition(mm) ± Standard Deviation(mm)]
25 µg	0.12±0.02	0.34±.009	0.14±0.03	1.28±0.15
50 µg	1.43±0.09	2.18±0.04	1.28±0.09	2.35±0.08
100 µg	4.27±0.03	3.48±0.14	2.97±0.05	5.36±0.17
Doxycycline	1.3±0.07	1.1±0.08	1.9±0.04	1.7±0.08

The mean width of the zones of inhibition around the microorganisms was compared using the paired t-test, and the results are shown in Table 2.

**Table 2:** The p-value of widths of the zones of inhibition around different microorganisms. Mean widths of the zone of inhibition obtained around different microorganisms which were compared using the paired t-test.

Organism	25 µg	50 µg	100 µg	Control
Streptococcus mutans				
Z	-0.843	-1.324	-2.481	-1.243
P-value	*0.008	*0.009	*0.017	*0.028
Lactobacillus				
Z	-1.346	-2.871	-4.283	-2.142
P-value	*0.028	*0.007	*0.032	*0.019
Enterococcus faecalis				
Z	-0.087	-1.273	-3.287	-1.275
P-value	*0.002	*0.012	*0.048	*0.016
Candida albicans				
Z	-1.428	-3.876	-5.472	-0.278
P-value	*0.027	*0.049	*0.036	*0.004

The study results indicated that concentrations of 50 µg and 100 µg of fenugreek gel showed greater antimicrobial activity against oral microbes compared with doxycycline. The zones of inhibition produced by fenugreek gel extract at these concentrations were greater when compared with those produced by doxycycline.

Anti-inflammatory activity

The mean and standard deviation of the fenugreek gel against aspirin with protein coagulation method in egg albumin was seen in Table 3. Paired t-test was used for statistical analysis and the P-value was less than 0.5 which indicates that the difference between the groups was statistically significant.

**Table 3:** The mean and standard deviation for the anti-inflammatory activity of fenugreek gel against aspirin. The mean and standard deviation for the anti-inflammatory activity of fenugreek gel against aspirin. Paired t-test was used for statistical analysis and the P-value was less than 0.5 which indicates that the difference between the groups was statistically significant.

Various concentrations (µg/ml)	Fenugreek extract (Mean inhibition ± Standard deviation)	Aspirin (Mean inhibition ± Standard deviation)	P- value
25 µg/ml	29.3±2.89	25.3±3.06	0.03
50 µg/ml	36.78±5.16	32.8±2.65	0.05
100 µg/ml	67.15±1.36	64.43±2.93	0.03

The fenugreek seed extract showed good anti-inflammatory activity with a maximum inhibition of 67.15 ± 1.36 observed at 100 µg/ml, and a standard anti-inflammatory drug (aspirin) showed a maximum inhibition of 64.43 ± 2.93 at the concentration of 100 µg/ml. The fenugreek extract has a higher potential to inhibit thermal denaturation of protein.

## Discussion

Periodontal disease and dental caries are caused by dental biofilm. While mechanical tooth brushing and dental flossing are important components of oral hygiene, periodontal packs also play a role in maintaining oral health because they prevent the exposure of the surgical site to oral bacteria [16]. The indiscriminate use of antibiotics and synthetic antibacterial agents has led to the emergence of multidrug-resistant microbial strains, and some microbial strains exhibit reduced susceptibility to antibiotics [17]. Natural therapeutic medications from various plant extracts have been developed to treat antimicrobial resistance [18].

Traditional plant-based medicines have demonstrated high effectiveness in providing antimicrobial compounds. Phytochemical compounds help combat various infections caused by microorganisms. Plants are known to be rich sources of secondary metabolites, including terpenoids, tannins, alkaloids, and flavonoids, and these secondary metabolites are often responsible for plants’ medicinal properties [19]. Agar well diffusion techniques were used to determine the effect of plant compounds in inhibiting the growth of specific microorganisms [20]. Both aqueous and methanol extracts of fenugreek seeds are found to have significant antibacterial activity against several bacterial strains, including *Pseudomonas* species, *Escherichia coli*, *Shigella dysenteriae*, and *Salmonella typhi* [21].

In another study, fenugreek sprout extract was found to have great antimicrobial properties. Fenugreek’s liquid form restricted the bacterial proliferation of *E. coli* and *Klebsiella pneumoniae* [22]. Different forms of fenugreek crude extracts did not inhibit Gram-negative bacteria, indicating that the antibacterial properties are not present in all forms of the extract and may be associated with specific chemical constituents [23]. Previous research found that the ethanolic chemical compounds extracted from fenugreek seeds were more effective than the aqueous compounds. Ethanolic compounds have a longer shelf life than aqueous compounds. Aqueous extracts are more susceptible to microbial contamination and degradation [24].

The study done for the anti-inflammatory activity test for petroleum extract of fenugreek gel showed that fenugreek gel showed a greater amount of anti-inflammatory activity when compared against diclofenac sodium [24]. The anti-inflammatory properties of fenugreek seed extract were evaluated by the anti-protein denaturalizing method of egg albumin. In the anti-denaturation assay, the denaturation of egg albumin was induced by heat treatment. The denatured protein expressed antigens associated with type III hypersensitive reactions and provoked delayed hypersensitivity [25]. This study’s results have shown considerable anti-inflammatory activity by fenugreek. It was capable of controlling the production of autoantigen and thereby inhibiting the denaturation of proteins and its effect was compared with aspirin. The secondary metabolites such as phenolic compounds and tannins, which were found in preliminary phytochemical screening, might be responsible for this activity.

Limitation of the study

The abovementioned study is an invitro study and, thus, has a low level of evidence.

## Conclusions

The study concludes that the fenugreek seed extract possesses higher antimicrobial and anti-inflammatory activity than standard antibiotic and anti-inflammatory drugs such as doxycycline and aspirin. Hence, the novel fenugreek gel could be used as an alternative periodontal dressing to reduce postoperative inflammation. However, further investigations are required for its application in vivo.
